# Supplementary material for: High GC content causes orphan proteins to be intrinsically disordered
Source: PLoS Comput Biol. 2017 Mar 29;13(3):e1005375. doi: 10.1371/journal.pcbi.1005375 (PMC5389847; doi:10.1371/journal.pcbi.1005375)
Supplement: S1 Table — (PDF) [file pcbi.1005375.s001.pdf]

Tax ID	Tax Name	Orphans	Genus Orphans	Intermediate	Ancient
936046	Agaricus bisporus var. bisporus h97	23 (0.24)%	0 (0.0%)	1075 (11.05%)	8633 (88.72%)
597362	Agaricus bisporus var. burnettii jb137-s8	40 (0.56)%	0 (0.0%)	832 (11.6%)	6299 (87.84%)
447093	Ajellomyces capsulatus g186ar	35 (0.41)%	27 (0.32%)	1245 (14.73%)	7148 (84.54%)
544712	Ajellomyces capsulatus h143	17 (0.19)%	343 (3.8%)	1311 (14.52%)	7359 (81.5%)
544711	Ajellomyces capsulatus h88	29 (0.32)%	343 (3.79%)	1360 (15.01%)	7329 (80.89%)
339724	Ajellomyces capsulatus nam1	71 (0.83)%	27 (0.32%)	583 (6.85%)	7829 (92.0%)
653446	Ajellomyces dermatitidis atcc 18188	22 (0.24)%	172 (1.85%)	1430 (15.38%)	7674 (82.53%)
559297	Ajellomyces dermatitidis er-3	12 (0.13)%	172 (1.85%)	1357 (14.59%)	7762 (83.44%)
559298	Ajellomyces dermatitidis slh14081	15 (0.16)%	0 (0.0%)	1549 (16.72%)	7699 (83.12%)
7167	Anopheles albimanus	0 (0.0)%	0 (0.0%)	1867 (17.54%)	8775 (82.46%)
7173	Anopheles arabiensis	0 (0.0)%	13 (0.11%)	2389 (19.77%)	9679 (80.12%)
41427	Anopheles atroparvus	0 (0.0)%	0 (0.0%)	2895 (22.8%)	9802 (77.2%)
43041	Anopheles christyi	0 (0.0)%	44 (0.43%)	1737 (16.92%)	8486 (82.65%)
139723	Anopheles culicifacies	0 (0.0)%	0 (0.0%)	3032 (22.59%)	10391 (77.41%)
43151	Anopheles darlingi	35 (0.35)%	0 (0.0%)	1362 (13.45%)	8733 (86.21%)
7168	Anopheles dirus	0 (0.0)%	0 (0.0%)	2109 (18.09%)	9548 (81.91%)
199890	Anopheles epiroticus	0 (0.0)%	0 (0.0%)	2065 (17.76%)	9561 (82.24%)
69004	Anopheles farauti	0 (0.0)%	0 (0.0%)	2800 (22.66%)	9555 (77.34%)
62324	Anopheles funestus	0 (0.0)%	0 (0.0%)	2197 (18.51%)	9674 (81.49%)
7165	Anopheles gambiae	73 (0.59)%	26 (0.21%)	1973 (15.82%)	10396 (83.38%)
74869	Anopheles maculatus	0 (0.0)%	0 (0.0%)	2913 (21.14%)	10869 (78.86%)
34690	Anopheles melas	0 (0.0)%	365 (2.38%)	3313 (21.59%)	11667 (76.03%)
30066	Anopheles merus	0 (0.0)%	13 (0.1%)	3260 (24.94%)	9799 (74.96%)
112268	Anopheles minimus	0 (0.0)%	0 (0.0%)	2135 (18.64%)	9317 (81.36%)
34691	Anopheles quadriannulatus	0 (0.0)%	20 (0.17%)	2386 (19.84%)	9618 (79.99%)
74873	Anopheles sinensis	513 (3.6)%	0 (0.0%)	3200 (22.46%)	10537 (73.94%)
30069	Anopheles stephensi	1 (0.01)%	5 (0.04%)	2161 (18.53%)	9496 (81.42%)
7462	Apis dorsata	0 (0.0)%	101 (0.52%)	2227 (11.44%)	17131 (88.04%)
7463	Apis florea	0 (0.0)%	134 (1.19%)	1920 (17.06%)	9199 (81.75%)
7460	Apis mellifera	946 (7.25)%	85 (0.65%)	2140 (16.41%)	9873 (75.69%)
663331	Arthroderma benhamiae cbs 112371	67 (0.88)%	0 (0.0%)	716 (9.37%)	6856 (89.75%)
535722	Arthroderma gypseum cbs 118893	53 (0.64)%	0 (0.0%)	699 (8.45%)	7519 (90.91%)
554155	Arthroderma otae cbs 113480	25 (0.3)%	0 (0.0%)	568 (6.93%)	7606 (92.77%)
344612	Aspergillus clavatus nrrl 1	35 (0.4)%	0 (0.0%)	642 (7.25%)	8182 (92.36%)
332952	Aspergillus flavus nrrl3357	64 (0.51)%	0 (0.0%)	1455 (11.63%)	10987 (87.85%)
451804	Aspergillus fumigatus a1163	10 (0.1)%	222 (2.27%)	793 (8.12%)	8747 (89.51%)
330879	Aspergillus fumigatus af293	3 (0.03)%	222 (2.32%)	754 (7.87%)	8604 (89.78%)
1033177	Aspergillus kawachii ifo 4308	13 (0.12)%	0 (0.0%)	1122 (10.0%)	10081 (89.88%)
227321	Aspergillus nidulans fgsc a4	164 (1.66)%	0 (0.0%)	631 (6.39%)	9081 (91.95%)
380704	Aspergillus niger atcc 1015	12 (0.11)%	108 (1.01%)	963 (8.98%)	9639 (89.9%)
425011	Aspergillus niger cbs 513.88	253 (2.15)%	108 (0.92%)	1214 (10.33%)	10176 (86.6%)
1160506	Aspergillus oryzae 3.042	0 (0.0)%	123 (1.08%)	1238 (10.9%)	9994 (88.01%)
510516	Aspergillus oryzae rib40	3 (0.03)%	123 (1.03%)	1357 (11.38%)	10441 (87.56%)
341663	Aspergillus terreus nih2624	31 (0.31)%	0 (0.0%)	754 (7.53%)	9227 (92.16%)
665024	Bipolaris maydis atcc 48331	10 (0.08)%	707 (5.64%)	1994 (15.92%)	9818 (78.36%)
701091	Bipolaris maydis c5	7 (0.06)%	708 (5.57%)	1979 (15.58%)	10011 (78.8%)
665912	Bipolaris sorokiniana nd90pr	66 (0.58)%	0 (0.0%)	1775 (15.5%)	9610 (83.92%)
132113	Bombus impatiens	0 (0.0)%	0 (0.0%)	2672 (21.48%)	9770 (78.52%)
30195	Bombus terrestris	0 (0.0)%	0 (0.0%)	2606 (21.36%)	9595 (78.64%)
1290391	Botryotinia fuckeliana bcdw1	13 (0.12)%	0 (0.0%)	1634 (15.14%)	9145 (84.74%)
999810	Botryotinia fuckeliana t4	802 (6.48)%	0 (0.0%)	1939 (15.68%)	9627 (77.84%)
135651	Caenorhabditis brenneri	545 (2.1)%	192 (0.74%)	7935 (30.55%)	17306 (66.62%)
6238	Caenorhabditis briggsae af16	470 (2.47)%	2220 (11.69%)	4576 (24.1%)	11724 (61.74%)
6239	Caenorhabditis elegans	229 (1.24)%	15 (0.08%)	6639 (36.01%)	11551 (62.66%)
281687	Caenorhabditis japonica	362 (1.57)%	17 (0.07%)	6127 (26.57%)	16551 (71.78%)
31234	Caenorhabditis remanei	214 (0.79)%	225 (0.83%)	8283 (30.59%)	18356 (67.79%)
237561	Candida albicans sc5314	250 (3.2)%	113 (1.44%)	914 (11.68%)	6546 (83.68%)
294748	Candida albicans wo-1	0 (0.0)%	105 (1.85%)	532 (9.35%)	5052 (88.8%)
573826	Candida dubliniensis cd36	2 (0.03)%	0 (0.0%)	539 (9.31%)	5246 (90.65%)
284593	Candida glabrata cbs 138	12 (0.24)%	0 (0.0%)	217 (4.29%)	4826 (95.47%)
1245528	Candida maltosa xu316	83 (1.45)%	0 (0.0%)	453 (7.94%)	5172 (90.61%)
1136231	Candida orthopsilosis co 90-125	4 (0.07)%	0 (0.0%)	494 (8.82%)	5105 (91.11%)
578454	Candida parapsilosis cdc317	2 (0.03)%	0 (0.0%)	504 (8.81%)	5213 (91.15%)
590646	Candida tenuis atcc 10573	19 (0.33)%	0 (0.0%)	372 (6.4%)	5425 (93.28%)
294747	Candida tropicalis mya-3404	22 (0.37)%	0 (0.0%)	563 (9.39%)	5411 (90.24%)
306901	Chaetomium globosum cbs 148.51	125 (1.25)%	0 (0.0%)	978 (9.78%)	8892 (88.96%)
759272	Chaetomium thermophilum var. thermophilum dsm 1495	49 (0.71)%	0 (0.0%)	437 (6.29%)	6462 (93.01%)
7719	Ciona intestinalis	759 (5.82)%	0 (0.0%)	1901 (14.57%)	10384 (79.61%)
51511	Ciona savignyi	182 (1.76)%	0 (0.0%)	1484 (14.37%)	8661 (83.87%)
246410	Coccidioides immitis rs	56 (0.64)%	0 (0.0%)	1132 (12.97%)	7539 (86.39%)
222929	Coccidioides posadasii c735 delta sowgp	5 (0.07)%	11 (0.15%)	382 (5.31%)	6793 (94.47%)
443226	Coccidioides posadasii str. silveira	93 (1.06)%	11 (0.13%)	1122 (12.84%)	7511 (85.97%)
1237896	Colletotrichum gloeosporioides cg-14	982 (6.38)%	184 (1.2%)	1894 (12.31%)	12324 (80.11%)
1213859	Colletotrichum gloeosporioides nara gc5	26 (0.17)%	185 (1.24%)	1856 (12.43%)	12865 (86.16%)
645133	Colletotrichum graminicola m1.001	73 (0.65)%	0 (0.0%)	1246 (11.12%)	9891 (88.23%)
759273	Colletotrichum higginsianum imi 349063	195 (1.41)%	0 (0.0%)	1579 (11.41%)	12069 (87.18%)
1213857	Colletotrichum orbiculare maff 240422	103 (0.84)%	0 (0.0%)	1482 (12.14%)	10620 (87.01%)
367775	Cryptococcus gattii wm276	28 (0.44)%	0 (0.0%)	767 (12.15%)	5519 (87.41%)
235443	Cryptococcus neoformans var. grubii h99	14 (0.2)%	0 (0.0%)	902 (12.66%)	6211 (87.15%)
283643	Cryptococcus neoformans var. neoformans b-3501a	0 (0.0)%	17 (0.26%)	778 (11.89%)	5747 (87.85%)
214684	Cryptococcus neoformans var. neoformans jec21	2 (0.03)%	17 (0.27%)	685 (10.8%)	5638 (88.9%)
7217	Drosophila ananassae	49 (0.35)%	0 (0.0%)	2871 (20.54%)	11056 (79.11%)
7220	Drosophila erecta	23 (0.16)%	35 (0.25%)	3237 (22.92%)	10828 (76.67%)
7222	Drosophila grimshawi	102 (0.72)%	0 (0.0%)	2780 (19.6%)	11302 (79.68%)
7227	Drosophila melanogaster	461 (3.29)%	19 (0.14%)	3008 (21.46%)	10528 (75.11%)
7230	Drosophila mojavensis	68 (0.5)%	0 (0.0%)	2668 (19.81%)	10735 (79.69%)
7234	Drosophila persimilis	124 (0.8)%	0 (0.0%)	3600 (23.3%)	11728 (75.9%)
7237	Drosophila pseudoobscura	6 (0.04)%	0 (0.0%)	3366 (22.46%)	11617 (77.5%)
7238	Drosophila sechellia	61 (0.4)%	673 (4.43%)	3026 (19.91%)	11437 (75.26%)
7240	Drosophila simulans	74 (0.52)%	35 (0.25%)	3523 (24.68%)	10644 (74.56%)

Continued on next page

Table S1 – *Continued from previous page*

Tax ID	Tax Name	Orphans	Genus Orphans	Intermediate	Ancient
7244	Drosophila virilis	46 (0.34)%	0 (0.0%)	2722 (20.02%)	10829 (79.64%)
7260	Drosophila willistoni	29 (0.2)%	0 (0.0%)	2589 (18.07%)	11707 (81.72%)
7245	Drosophila yakuba	32 (0.22)%	10 (0.07%)	3422 (23.09%)	11357 (76.63%)
284813	Encephalitozoon cuniculi gb-m1	5 (0.26)%	0 (0.0%)	379 (19.72%)	1538 (80.02%)
907965	Encephalitozoon hellem atcc 50504	0 (0.0)%	0 (0.0%)	367 (20.23%)	1447 (79.77%)
876142	Encephalitozoon intestinalis atcc 50506	3 (0.17)%	0 (0.0%)	367 (20.19%)	1448 (79.65%)
1178016	Encephalitozoon romaleae sj-2008	0 (0.0)%	0 (0.0%)	370 (20.37%)	1446 (79.63%)
1279085	Fusarium fujikuroi imi 58289	27 (0.19)%	0 (0.0%)	1972 (13.8%)	12295 (86.02%)
229533	Fusarium graminearum ph-1	133 (1.08)%	0 (0.0%)	1688 (13.76%)	10448 (85.16%)
1229664	Fusarium oxysporum f. sp. cubense race 1	5 (0.03)%	96 (0.64%)	2308 (15.29%)	12681 (84.04%)
1229665	Fusarium oxysporum f. sp. cubense race 4	4 (0.03)%	96 (0.69%)	2115 (15.21%)	11693 (84.07%)
426428	Fusarium oxysporum f. sp. lycopersici 4287	393 (2.43)%	0 (0.0%)	2308 (14.28%)	13459 (83.29%)
660025	Fusarium oxysporum fo5176	240 (1.42)%	44 (0.26%)	2234 (13.24%)	14353 (85.07%)
1028729	Fusarium pseudograminearum cs3096	112 (0.93)%	0 (0.0%)	1555 (12.94%)	10347 (86.12%)
1104152	Glarea lozoyensis 74030	243 (3.45)%	0 (0.0%)	731 (10.39%)	6063 (86.16%)
1116229	Glarea lozoyensis atcc 20868	110 (0.92)%	0 (0.0%)	1339 (11.18%)	10525 (87.9%)
1071382	Kazachstania africana cbs 2517	7 (0.13)%	0 (0.0%)	249 (4.73%)	5004 (95.13%)
1071383	Kazachstania naganishii cbs 8797	9 (0.17)%	0 (0.0%)	227 (4.37%)	4960 (95.46%)
981350	Komagataella pastoris cbs 7435	5 (0.1)%	0 (0.0%)	277 (5.61%)	4656 (94.29%)
644223	Komagataella pastoris gs115	1 (0.02)%	0 (0.0%)	271 (5.43%)	4722 (94.55%)
242507	Magnaporthe oryzae 70-15	56 (0.47)%	89 (0.75%)	2479 (20.87%)	9257 (77.91%)
1143193	Magnaporthe oryzae p131	61 (0.5)%	675 (5.51%)	2450 (20.0%)	9062 (73.99%)
1143189	Magnaporthe oryzae y34	74 (0.6)%	88 (0.71%)	3068 (24.83%)	9126 (73.86%)
644358	Magnaporthe poae atcc 64411	189 (1.95)%	0 (0.0%)	1247 (12.88%)	8246 (85.17%)
425265	Malassezia globosa cbs 7966	18 (0.43)%	0 (0.0%)	215 (5.16%)	3935 (94.41%)
1230383	Malassezia sympodialis atcc 42132	0 (0.0)%	0 (0.0%)	141 (4.2%)	3217 (95.8%)
655827	Metarhizium acridum cqma 102	4 (0.04)%	0 (0.0%)	829 (8.67%)	8729 (91.29%)
655844	Metarhizium anisopliae arsef 23	28 (0.27)%	0 (0.0%)	922 (8.88%)	9429 (90.85%)
554373	Moniliophthora perniciosa fa553	63 (0.55)%	0 (0.0%)	1200 (10.57%)	10091 (88.88%)
1381753	Moniliophthora roreri mca 2997	82 (0.51)%	0 (0.0%)	1613 (10.06%)	14346 (89.43%)
1064592	Naumovozyma castellii cbs 4309	10 (0.18)%	0 (0.0%)	275 (5.02%)	5191 (94.8%)
1071378	Naumovozyma dairenensis cbs 421	11 (0.2)%	0 (0.0%)	248 (4.55%)	5186 (95.24%)
881290	Nematocida parisii ertm1	2 (0.08)%	146 (5.64%)	645 (24.92%)	1795 (69.36%)
935791	Nematocida parisii ertm3	11 (0.42)%	147 (5.56%)	655 (24.77%)	1831 (69.25%)
944018	Nematocida sp. 1 ertm2	21 (0.84)%	0 (0.0%)	657 (26.21%)	1829 (72.96%)
367110	Neurospora crassa or74a	20 (0.2)%	0 (0.0%)	1659 (16.86%)	8162 (82.94%)
510951	Neurospora tetrasperma fgsc 2508	14 (0.14)%	766 (7.57%)	1696 (16.76%)	7644 (75.53%)
510952	Neurospora tetrasperma fgsc 2509	56 (0.54)%	766 (7.37%)	1774 (17.06%)	7801 (75.03%)
578461	Nosema bombycis cq1	75 (2.03)%	0 (0.0%)	404 (10.95%)	3210 (87.02%)
578460	Nosema ceranae brl01	10 (0.53)%	0 (0.0%)	213 (11.21%)	1677 (88.26%)
482561	Paracoccidioides brasiliensis pb03	11 (0.15)%	193 (2.56%)	663 (8.8%)	6669 (88.5%)
502780	Paracoccidioides brasiliensis pb18	11 (0.14)%	193 (2.44%)	760 (9.63%)	6932 (87.79%)
502779	Paracoccidioides sp. 'lutzii' pb01	51 (0.65)%	0 (0.0%)	817 (10.47%)	6936 (88.88%)
500485	Penicillium chrysogenum wisconsin 54-1255	63 (0.55)%	0 (0.0%)	1122 (9.81%)	10252 (89.64%)
1170230	Penicillium digitatum pd1	2 (0.02)%	411 (4.64%)	785 (8.86%)	7663 (86.48%)
1170229	Penicillium digitatum phi26	2 (0.02)%	411 (4.57%)	788 (8.75%)	7800 (86.66%)
1209962	Pneumocystis jirovecii se8	14 (0.44)%	0 (0.0%)	82 (2.58%)	3080 (96.98%)
1069680	Pneumocystis murina b123	6 (0.16)%	0 (0.0%)	83 (2.26%)	3583 (97.58%)
1151754	Pseudozyma antarctica t-34	38 (0.6)%	0 (0.0%)	874 (13.7%)	5466 (85.7%)
1305764	Pseudozyma hubeiensis sy62	74 (1.16)%	0 (0.0%)	905 (14.19%)	5400 (84.65%)
418459	Puccinia graminis f. sp. tritici crl 75-36-700-3	202 (1.57)%	0 (0.0%)	1067 (8.31%)	11565 (90.11%)
630390	Puccinia triticina 1-1 bbbd race 1	161 (1.54)%	0 (0.0%)	794 (7.57%)	9533 (90.89%)
861557	Pyrenophora teres f. teres 0-1	54 (0.49)%	0 (0.0%)	1586 (14.38%)	9386 (85.13%)
426418	Pyrenophora tritici-repentis pt-1c-bfp	85 (0.77)%	0 (0.0%)	1614 (14.63%)	9332 (84.6%)
983506	Rhizoctonia solani ag-1 ia	265 (3.4)%	0 (0.0%)	684 (8.77%)	6849 (87.83%)
1108050	Rhizoctonia solani ag-1 ib	166 (1.48)%	0 (0.0%)	829 (7.41%)	10189 (91.1%)
1160507	Saccharomyces arboricola h-6	0 (0.0)%	0 (0.0%)	173 (4.76%)	3464 (95.24%)
545124	Saccharomyces cerevisiae awri1631	132 (2.57)%	0 (0.0%)	326 (6.35%)	4676 (91.08%)
764097	Saccharomyces cerevisiae awri796	1 (0.03)%	5 (0.13%)	243 (6.5%)	3491 (93.34%)
889517	Saccharomyces cerevisiae cen.pk113-7d	0 (0.0)%	2 (0.04%)	309 (5.72%)	5091 (94.24%)
643680	Saccharomyces cerevisiae ec1118	0 (0.0)%	0 (0.0%)	823 (14.08%)	5022 (85.92%)
764102	Saccharomyces cerevisiae fostersb	3 (0.08)%	0 (0.0%)	229 (6.25%)	3433 (93.67%)
764101	Saccharomyces cerevisiae fosterso	4 (0.11)%	0 (0.0%)	227 (6.46%)	3285 (93.43%)
574961	Saccharomyces cerevisiae jay291	1 (0.02)%	0 (0.0%)	361 (6.99%)	4801 (92.99%)
721032	Saccharomyces cerevisiae kyokai no. 7	1 (0.02)%	1 (0.02%)	409 (7.2%)	5273 (92.77%)
764098	Saccharomyces cerevisiae lalvin qa23	1 (0.03)%	4 (0.1%)	244 (6.14%)	3725 (93.73%)
285006	Saccharomyces cerevisiae rm11-1a	3 (0.06)%	0 (0.0%)	384 (7.23%)	4926 (92.72%)
559292	Saccharomyces cerevisiae s288c	16 (0.25)%	5 (0.08%)	909 (14.06%)	5536 (85.62%)
764099	Saccharomyces cerevisiae vin13	1 (0.03)%	1 (0.03%)	247 (6.35%)	3640 (93.6%)
764100	Saccharomyces cerevisiae vl3	0 (0.0)%	5 (0.13%)	266 (6.67%)	3719 (93.21%)
307796	Saccharomyces cerevisiae yjm789	0 (0.0)%	2 (0.03%)	411 (7.08%)	5391 (92.88%)
226230	Saccharomyces kudriavzevii ifo 1802	0 (0.0)%	0 (0.0%)	231 (6.22%)	3480 (93.78%)
653667	Schizosaccharomyces cryophilus oy26	13 (0.26)%	0 (0.0%)	423 (8.48%)	4554 (91.26%)
402676	Schizosaccharomyces japonicus yfs275	16 (0.34)%	0 (0.0%)	315 (6.73%)	4350 (92.93%)
483514	Schizosaccharomyces octosporus yfs286	5 (0.1)%	0 (0.0%)	400 (8.18%)	4484 (91.72%)
284812	Schizosaccharomyces pombe 972h-	18 (0.36)%	0 (0.0%)	389 (7.88%)	4530 (91.76%)
936435	Serpula lacrymans var. lacrymans s7.3	88 (0.67)%	0 (0.0%)	2295 (17.56%)	10684 (81.76%)
578457	Serpula lacrymans var. lacrymans s7.9	73 (0.62)%	0 (0.0%)	2201 (18.65%)	9528 (80.73%)
441960	Talaromyces marneffei atcc 18224	11 (0.11)%	0 (0.0%)	648 (6.39%)	9484 (93.5%)
441959	Talaromyces stipitatus atcc 10500	11 (0.09)%	0 (0.0%)	813 (6.57%)	11545 (93.34%)
1071380	Tetrapisispora blattae cbs 6284	17 (0.32)%	0 (0.0%)	236 (4.47%)	5027 (95.21%)
1071381	Tetrapisispora phaffii cbs 4417	11 (0.21)%	0 (0.0%)	251 (4.88%)	4877 (94.9%)
452589	Trichoderma atroviride imi 206040	96 (0.89)%	0 (0.0%)	1056 (9.77%)	9661 (89.35%)
431241	Trichoderma reesei qm6a	36 (0.41)%	0 (0.0%)	774 (8.79%)	8000 (90.81%)
413071	Trichoderma virens gv29-8	40 (0.34)%	0 (0.0%)	1233 (10.57%)	10387 (89.08%)
559882	Trichophyton equinum cbs 127.97	24 (0.29)%	0 (0.0%)	1025 (12.37%)	7240 (87.34%)
559305	Trichophyton rubrum cbs 118892	40 (0.5)%	0 (0.0%)	755 (9.41%)	7227 (90.09%)
647933	Trichophyton tonsurans cbs 112818	14 (0.17)%	0 (0.0%)	995 (12.07%)	7235 (87.76%)
663202	Trichophyton verrucosum hki 0517	63 (0.82)%	0 (0.0%)	730 (9.53%)	6869 (89.65%)
1186058	Trichosporon asahii var. asahii cbs 2479	23 (0.28)%	0 (0.0%)	1059 (13.09%)	7009 (86.63%)

Continued on next page

Table S1 – *Continued from previous page*

Tax ID	Tax Name	Orphans	Genus Orphans	Intermediate	Ancient
1220162	Trichosporon asahii var. asahii cbs 8904	33 (0.4)%	0 (0.0%)	1062 (12.88%)	7150 (86.72%)
1128400	Ustilago hordei uh4857-4	28 (0.41)%	0 (0.0%)	854 (12.6%)	5897 (86.99%)
237631	Ustilago maydis 521	15 (0.24)%	0 (0.0%)	824 (13.28%)	5364 (86.47%)
526221	Verticillium alfalfae vams.102	51 (0.52)%	0 (0.0%)	968 (9.92%)	8737 (89.56%)
498257	Verticillium dahliae vdls.17	53 (0.53)%	0 (0.0%)	998 (9.94%)	8990 (89.53%)
1299270	Wallemia ichthyophaga exf-994	14 (0.3)%	0 (0.0%)	247 (5.28%)	4419 (94.42%)
671144	Wallemia sebi cbs 633.66	7 (0.14)%	0 (0.0%)	263 (5.2%)	4784 (94.66%)
