# Supplementary material for: High GC content causes orphan proteins to be intrinsically disordered
Source: PLoS Comput Biol. 2017 Mar 29;13(3):e1005375. doi: 10.1371/journal.pcbi.1005375 (PMC5389847; doi:10.1371/journal.pcbi.1005375)
Supplement: S2 Table — (PDF) [file pcbi.1005375.s002.pdf]

AA	GC%	TOP-IDP	Hessa	Alpha	Beta	Coil	Turn
Ala	0.83	0.06	0.11	0.17	-0.15	-0.08	-0.10
Arg	0.72	0.18	2.58	0.09	-0.04	-0.05	-0.04
Asn	0.17	0.01	2.05	-0.11	-0.22	0.07	0.20
Asp	0.50	0.19	3.49	-0.03	-0.27	0.08	0.08
Cys	0.50	0.02	-0.13	-0.02	0.08	-0.02	-0.02
Gln	0.50	0.32	2.36	0.07	-0.08	-0.02	0.00
Glu	0.50	0.74	2.68	0.18	-0.25	-0.12	0.06
Gly	0.83	0.17	0.74	-0.29	-0.18	0.10	0.27
His	0.50	0.30	2.06	0.00	-0.06	0.03	-0.01
Ile	0.11	-0.49	-0.60	0.00	0.26	-0.05	-0.62
Leu	0.39	-0.33	-0.55	0.09	0.10	-0.09	-0.17
Lys	0.17	0.59	2.71	0.07	-0.14	-0.05	0.11
Met	0.33	-0.40	-0.10	0.13	0.08	-0.09	-0.36
Phe	0.17	-0.70	-0.32	0.08	0.14	-0.10	-0.20
Pro	0.83	0.99	2.23	-0.31	-0.45	0.19	0.15
Ser	0.50	0.34	0.84	-0.13	-0.03	0.05	0.12
Thr	0.50	0.06	0.52	-0.11	0.09	0.06	-0.13
Trp	0.67	-0.88	0.30	0.04	0.12	-0.03	-0.26
Tyr	0.17	-0.51	0.68	-0.10	0.10	0.04	-0.10
Val	0.50	-0.12	-0.31	0.00	0.29	-0.11	-0.41

Table S2: For each amino acid we show the GC content of its codons, its value in the TOP-IDP scale, its hydrophobicity (Hessa scale), as well as its value in the secondary structure scales for Alpha, Beta, Coil and Turn propensity.
