# Supplementary figures and images for: High GC content causes orphan proteins to be intrinsically disordered
Source: PLoS Comput Biol. 2017 Mar 29;13(3):e1005375. doi: 10.1371/journal.pcbi.1005375 (PMC5389847; doi:10.1371/journal.pcbi.1005375)

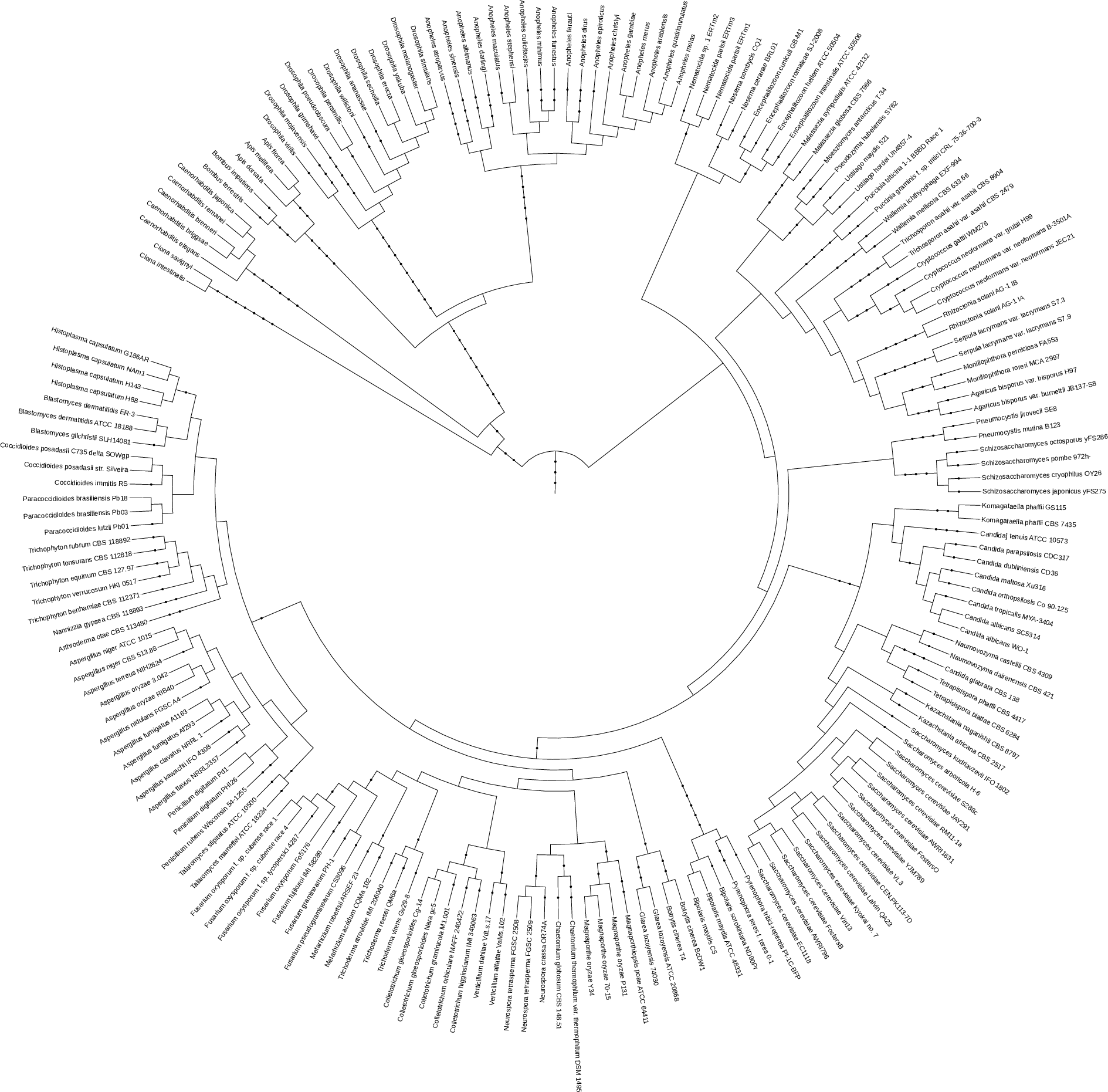

Supplement: S1 Fig — (TIF) [file pcbi.1005375.s003.tif]

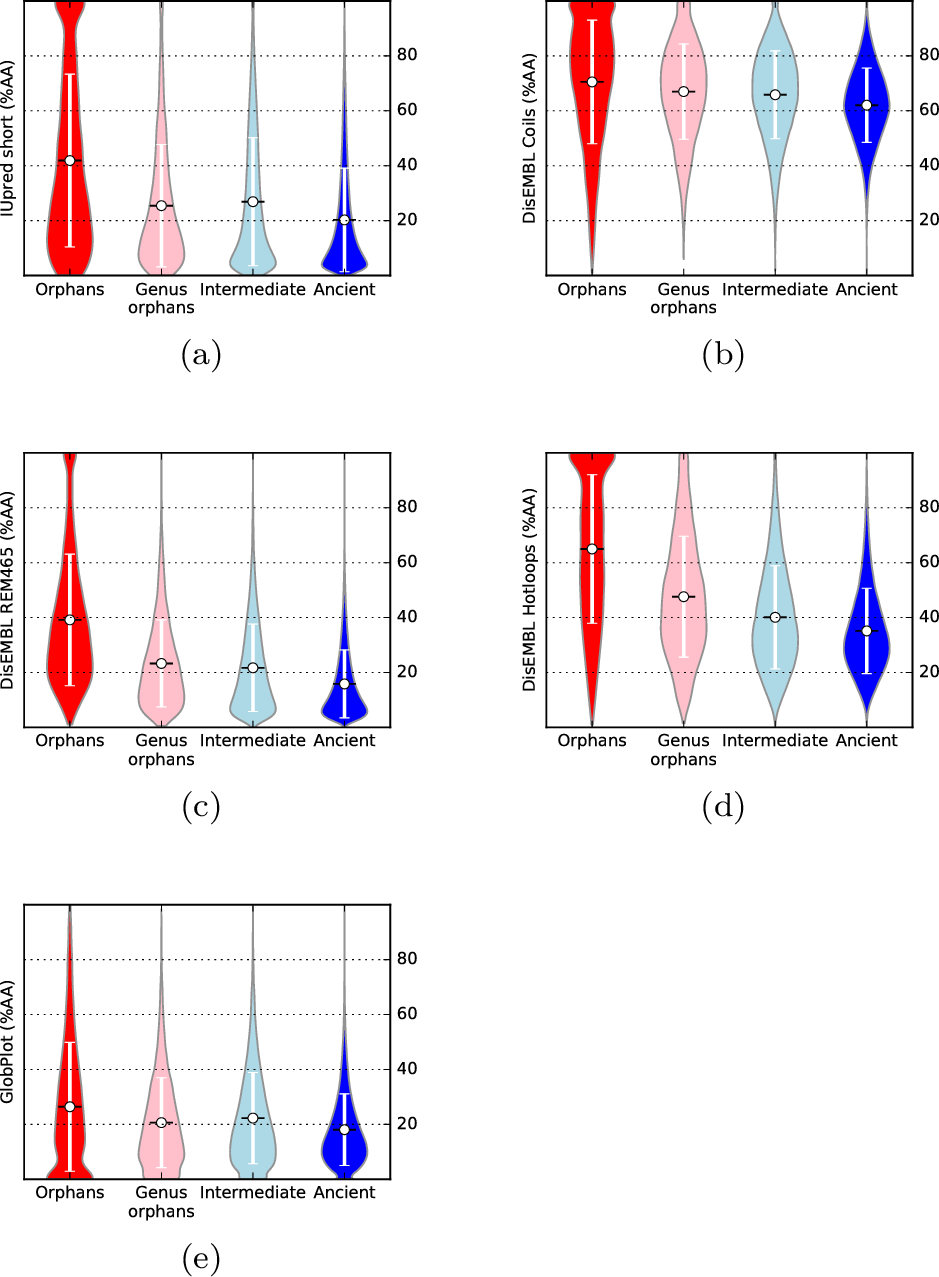

Supplement: S2 Fig — (TIF) [file pcbi.1005375.s004.tif]

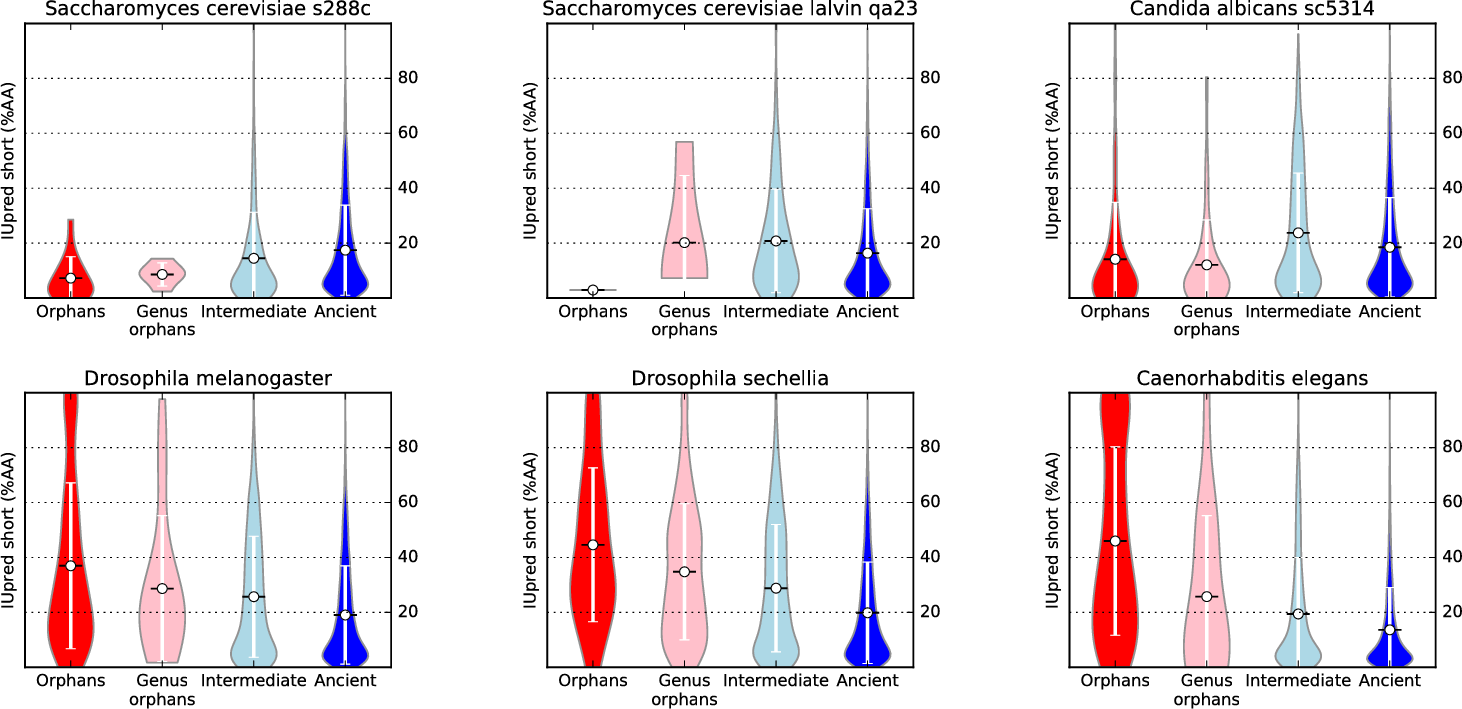

Supplement: S3 Fig — (TIF) [file pcbi.1005375.s005.tif]

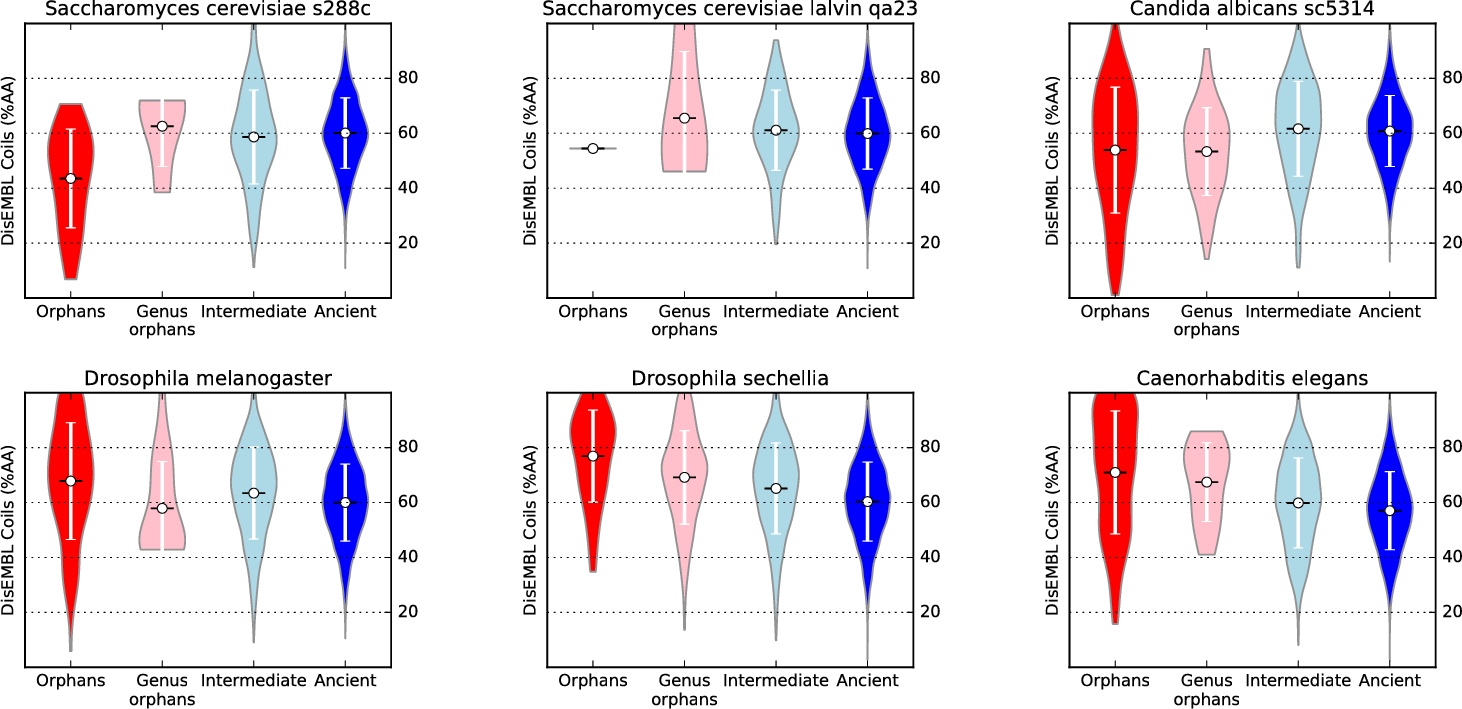

Supplement: S4 Fig — (TIF) [file pcbi.1005375.s006.tif]

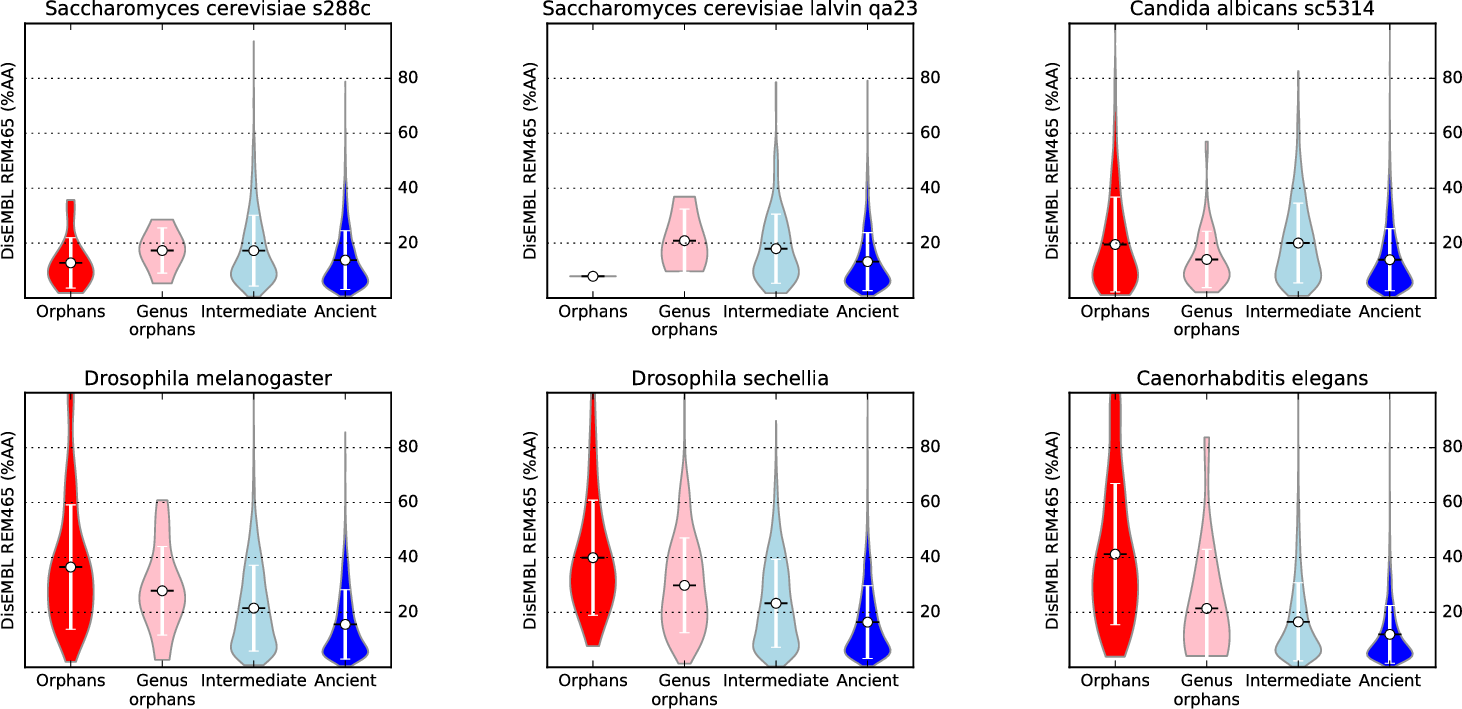

Supplement: S5 Fig — (TIF) [file pcbi.1005375.s007.tif]

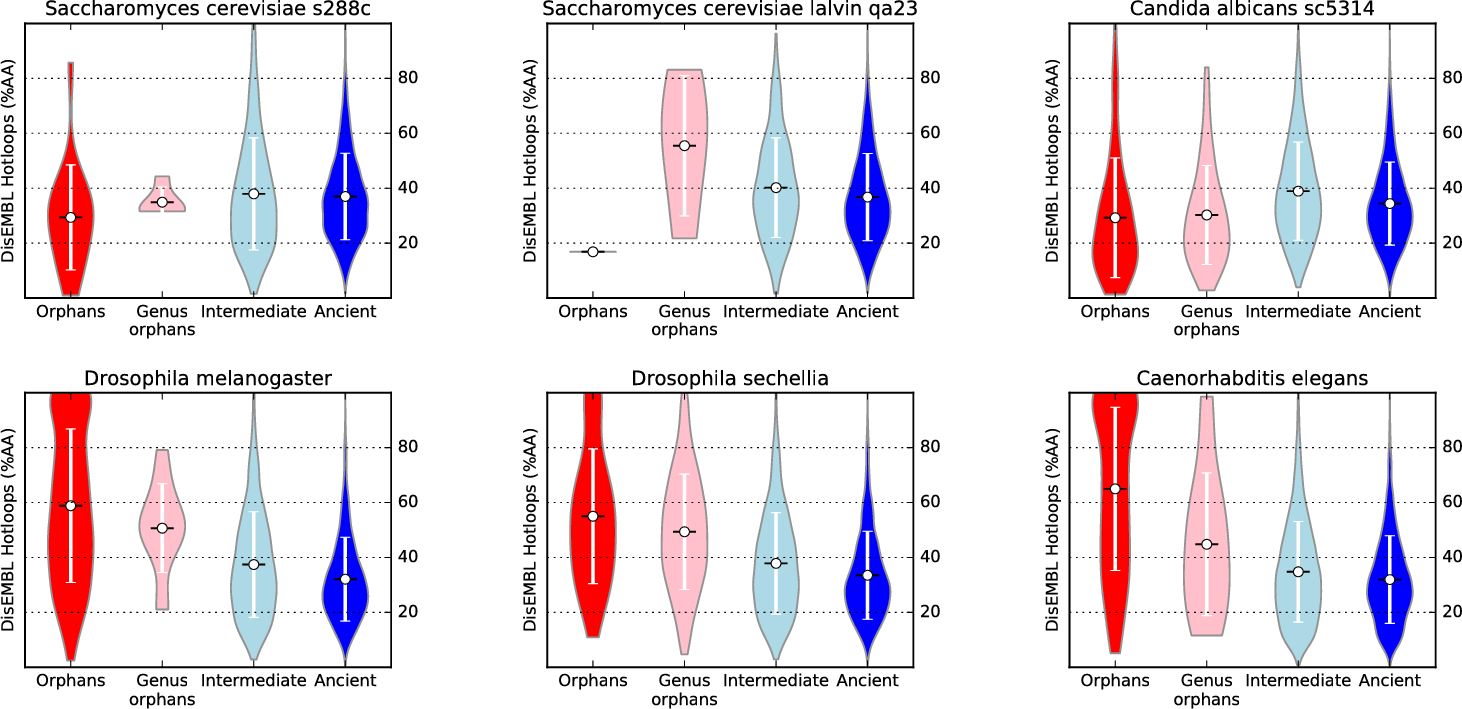

Supplement: S6 Fig — (TIF) [file pcbi.1005375.s008.tif]

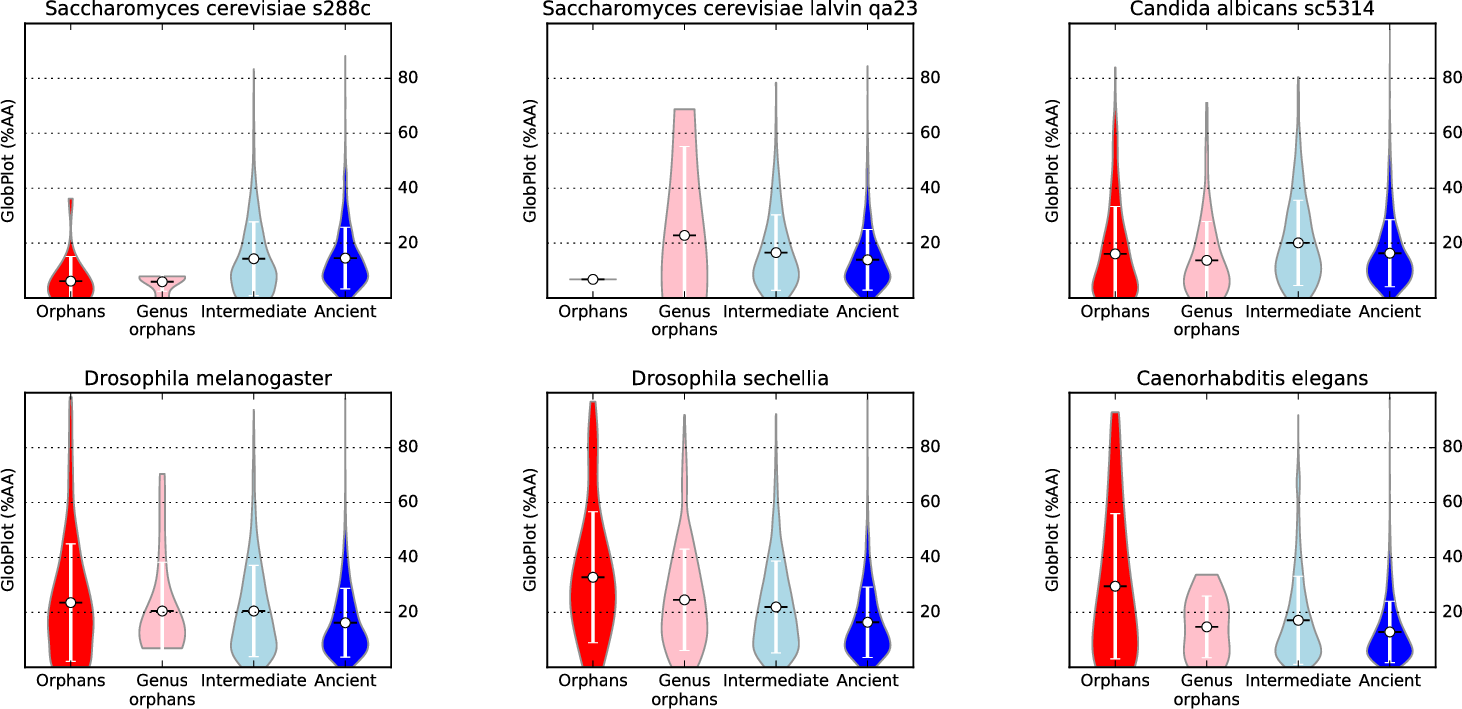

Supplement: S7 Fig — (TIF) [file pcbi.1005375.s009.tif]

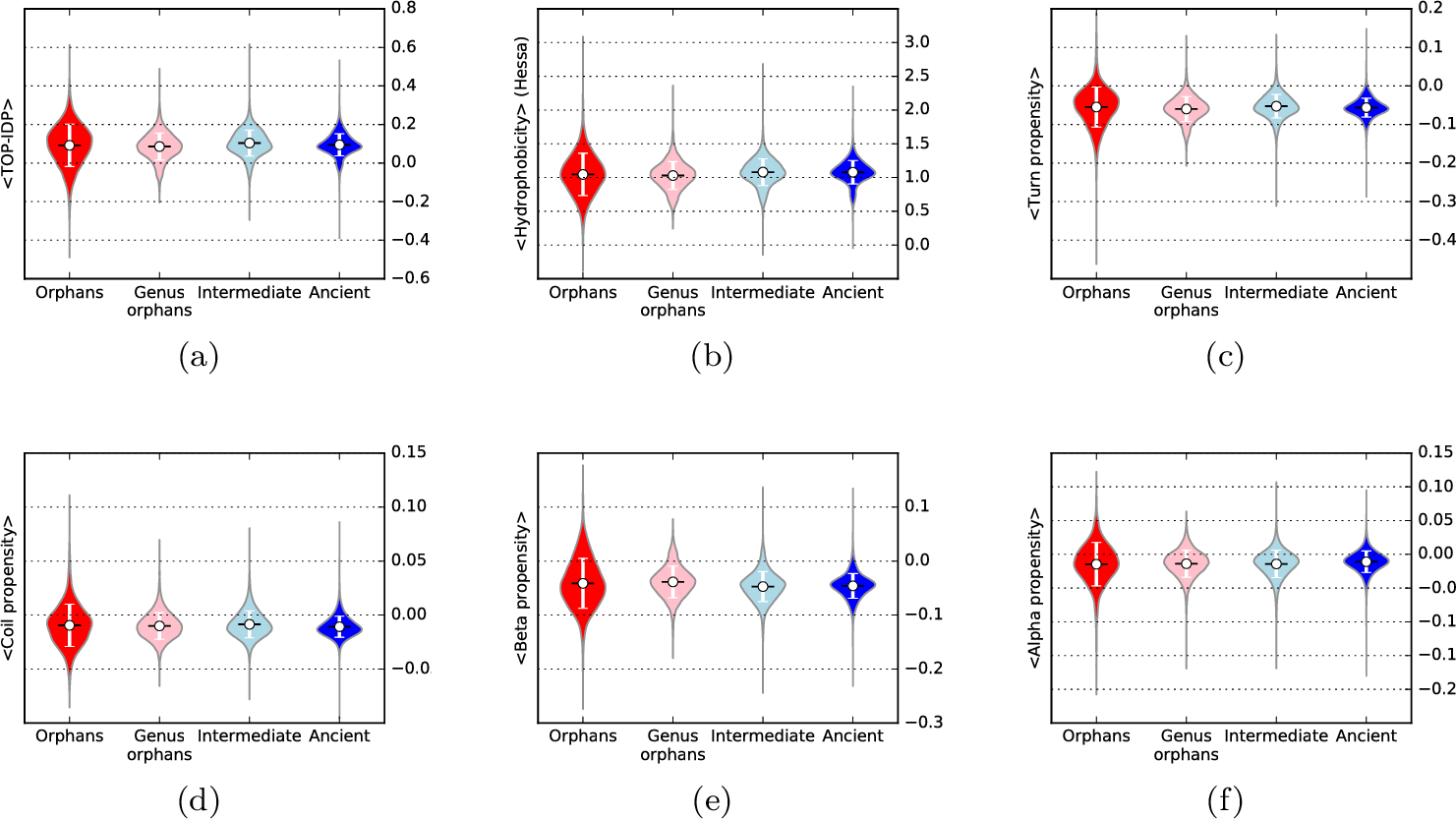

Supplement: S8 Fig — (a) Intrinsic disorder using the TOP-IDP scale, (b) hydrophobicity using the Hessa scale, (c-f) secondary structure preferences. (TIF) [file pcbi.1005375.s010.tif]
